# The Effect of Copper and Zinc Sources on Liver Copper and Zinc Concentrations and Performance of Beef Cows and Suckling Calves

**DOI:** 10.3390/vetsci10080511

**Published:** 2023-08-08

**Authors:** Jesse O. Fulton, Amanda D. Blair, Keith R. Underwood, Russell F. Daly, Michael G. Gonda, George A. Perry, Cody L. Wright

**Affiliations:** 1 Department of Animal Science, University of Nebraska Panhandle Research & Extension Center, 4502 Ave I, Scottsbluff, NE 69361, USA; 2 Department of Animal Science, South Dakota State University, P.O. Box 2170, Brookings, SD 57007, USA; amanda.blair@sdstate.edu (A.D.B.); keith.underwood@sdstate.edu (K.R.U.); michael.gonda@sdstate.edu (M.G.G.); cody.wright@sdstate.edu (C.L.W.); 3 Department of Veterinary and Biomedical Sciences, South Dakota State University, P.O. Box 2175, Brookings, SD 57007, USA; russell.daly@sdstate.edu; 4 Department of Animal Science, Texas A&M AgriLife Research and Extension Center, 1710 FM3053, Overton, TX 75684, USA; george.perry@ag.tamu.edu

**Keywords:** beef cattle, copper, gestation, organic mineral, zinc

## Abstract

**Simple Summary:**

During gestation, increased nutrient intake is required by the cow to meet the requirements of the maturing fetus, and if these requirements are not met, changes in fetal development can occur. However, little is understood about the effects of gestational manipulation of minerals on progeny growth and immune function. Minerals provided in an organic form have been shown to be more soluble and readily available to the body for absorption. Therefore, the objectives of this study are to determine if the source of gestational copper and zinc affects cow reproductive performance, calf growth, and calf health. Cows (*n* = 287) were assigned to an inorganic or organic source of copper and zinc prior to breeding, and the treatments continued until weaning. Cow body weight, pregnancy data, calf weaning weight, and antibody response were recorded. The cows receiving the inorganic treatment had heavier body weights and body condition scores at breeding in Year 2 of the study, while the cows on the organic treatment had heavier body weights at weaning in Year 2. The cows that received the organic mineral had improved pregnancy rates in Year 1. The calves from the organic treatment had heavier weaning weights but there was no influence on calf health.

**Abstract:**

To determine the influence of the source of gestational and postnatal Cu and Zn supplementation on cow and calf performance, cows (*n* = 287) were assigned to one of the following two treatments: (1) inorganic (INORG) treatment, in which cows were supplemented with 15 mg of Cu (as CuSO_4_) and 15 mg of Zn (as ZnSO_4_) per kg of diet DM, or (2) organic (ORG) treatment, in which cows were supplemented with 15 mg of Cu (as Cu proteinate; Bioplex Cu, Alltech, Inc., Nicholasville, KY, USA) and 15 mg of Zn (as Zn proteinate; Bioplex Zn, Alltech, Inc., Nicholasville, KY, USA) per kg of diet DM. The treatments were initiated prior to breeding and continued throughout gestation until weaning. Liver biopsies were collected for analysis of mineral content. Cow body condition score (BCS), body weight (BW), pregnancy data, calf weaning weight (WW), and antibody response of the calves were recorded. The cows receiving the INORG treatment had a greater BW (*p* < 0.05) and BCS *(p <* 0.01) at breeding in Year 2, while the cows on the ORG treatment had a greater (*p <* 0.05) BW at weaning in Year 2. The cows that received the ORG mineral had improved (*p <* 0.05) conception rates in Year 1. The calves receiving the ORG treatment had heavier (*p <* 0.05) 205-day adjusted WWs.

## 1. Introduction

It is understood that during gestation, cows must increase their nutrient intake to meet the requirements of a maturing fetus. Previous research has demonstrated that if these requirements are not met, changes in fetal development can occur [1,2,3,4]. The bioavailability of supplemental trace mineral source in ruminant diets can be highly variable. Sulfate and chloride sources are generally considered the most available for absorption, but the bioavailability of oxide sources depends on the element. Zinc oxide is considered to have a similar bioavailability to ZnSO_4_; however, CuO in powder form is essentially unavailable to ruminants [5]. Research has demonstrated that organic forms of trace minerals are more soluble and readily available to the body for absorption [6,7,8].

Copper is an essential component of numerous enzymes that support key physiological processes. Unfortunately, Cu deficiency is a widespread issue in many parts of North America and can lead to many negative effects, including impaired reproduction [9]. Zinc is highly ubiquitous in physiological systems. It is required for the structural and functional integrity of over 2000 transcription factors and is a component of zinc finger-binding domains in DNA-binding proteins [10]. In forages, Cu and Zn are commonly found in concentrations that are at least marginally deficient [11], and Cu is subject to very potent antagonisms from S, Mo, and Fe, further reducing the amount that is available to the animal [9]. Supplementation with more bioavailable forms of Cu and Zn may result in enhanced mineral status and potentially affect animal productivity. Therefore, the objectives of this study were to determine the influence of Cu and Zn sources on the reproductive performance of cows and the health and pre-weaning growth of calves.

## 2. Materials and Methods

### 2.1. Animals

The South Dakota State University Institutional Animal Care and Use Committee approved all animal procedures before project initiation (IACUC approval #12-035A). Commercial cows, primarily of Angus genetics (*n* = 287), located at the South Dakota State University Antelope Range and Livestock Research Station (45.515346, −103.311560) were assigned to treatments based on cow age. Groups were randomly assigned to the following two supplemental mineral treatments: (1) inorganic (INORG) treatment, in which cows were supplemented with 15 mg of Cu and 15 of mg Zn per kg of total diet DM in the form of CuSO_4_ and ZnSO_4_, respectively, and (2) organic (ORG) treatment, in which cows were supplemented with 15 mg of Cu and 15 mg of Zn per kg of total diet DM in a proteinated form (Bioplex Cu 10% and Bioplex Zn 15%, Alltech Inc., Nicholasville, KY, USA). All other supplemented minerals were provided in their inorganic form. Prior to formulation of the mineral supplements, forage and water samples were collected from dormant pastures that the cattle would be subsequently grazing. Mineral supplements were formulated to meet or exceed the NRC requirement for each mineral. However, Cu was supplemented to provide 3 times the NRC requirement to account for the antagonism created by the concentrations of Mo in the forages (range = 0.98 to 4.35 mg Mo/kg DM) and SO_4_ in the water (range = 5.1 to 5100 mg SO_4_/L). Cows began to receive their assigned mineral supplement on 26 May of Year 1, 30 days prior to timed artificial insemination (AI) in Year 1. Treatment groups were maintained in separate pastures and received their treatments via free-choice loose mineral supplement. Intake of the mineral supplement was monitored weekly and, if necessary, white salt was incorporated as an intake modifier to maintain intake of the mineral supplement at approximately 85.05 g/head/day. Cows and calves remained on treatment supplementation until weaning in October of Year 2. At weaning, 245 calves and 239 cows remained on the trial due to normal culling and death loss. Cow body condition score (BCS, measured from 1 to 9; 1 = extremely emaciated, 9 = very obese [12]) and body weights (BWs) were recorded at trial initiation (May of Year 1), weaning (October of Year 1; these calves were not part of the study), at the time of breeding, (June of Year 2), and at weaning of the calves in the study (October of Year 2). An additional BW was recorded in December of Year 1. Calf weights were collected at birth and at weaning.

During the grazing season, cattle were maintained on a rotational grazing system. Forage samples were collected prior to each group of cattle entering each pasture rotation throughout the duration of the project. Cows were turned out to pasture on 7 June of Year 1 and 28 May of Year 2.

From the time they were turned out on pasture until 30 November, the cattle only received their mineral supplement. No additional feeds were provided. Beginning on 1 December, the cows were fed a 30% crude protein commercial supplement at a rate of 1.36 kg every other day while the cows grazed on pasture. Cows continued to graze pasture and receive both the commercial crude protein supplement and their mineral supplement until approximately 1 week prior to calving. As each individual cow began to display visual signs of impending parturition, they were relocated to one of two drylot pens based on their treatment group. While in the drylot pens, cows had ad libitum access to hay from one of two sources and were fed 0.68 kg of the commercial crude protein supplement per animal per day. Mineral supplements were provided ad libitum to each of the appropriate treatment groups while in the drylot pens. Approximately 1 day after parturition, each cow–calf pair was relocated back to a pasture where they had ad libitum access to their appropriate mineral supplement and were fed 1.36 kg of the commercial crude protein supplement every other day. Feeding of the commercial crude protein supplement was discontinued when the cattle were turned out on new pastures on 28 May of Year 2.

Throughout the experiment, feed and forage samples were collected, stored, and pooled prior to analysis. Feeds and forages were analyzed for DM, CP, ADF, NDF, and a mineral panel. Water was analyzed for SO_4_ prior to the initiation of the experiment.

Forage nutrient composition, hay and winter crude protein supplement composition, and mineral supplement composition are presented in Table 1, Table 2 and Table 3, respectively. Estimated Cu and Zn intake and diet concentration for cows at various time points throughout the experiment are shown in Table 4.

Pasture samples were collected when cows were rotated into that specific pasture. Individual pasture sample results were averaged and reported as a single year for each treatment. It should be noted that in Year 1, the region was experiencing drought conditions. The average annual precipitation over the 50 years prior to this experiment being conducted is 39.5 cm [13]. During this experiment, the total precipitation for the area during the time this study was conducted was 27.58 cm for Year 1 (January–December), and 43.87 cm for Year 2 (January–October).

### 2.2. Analysis of Liver Mineral Content

Liver samples were collected from a randomly selected subsample of both cows and calves to determine change in Cu and Zn liver concentration over the treatment period. The cows and calves that were selected for liver biopsy were the same animals biopsied throughout the trial. Liver samples were collected using the True-Cut technique described by Pearson and Craig [14], as modified by Engle and Spears [15]. Samples were frozen at −20 °C until analysis. Initially, 88 cows were randomly selected from each treatment for liver biopsies. Cows were biopsied a total of 4 times as follows: (1) May of Year 1, immediately prior to initiation of the supplementation period to determine baseline mineral status; (2) December of Year 1, at the time the cows were moved off pasture and began receiving hay; (3) May of Year 2, when cows were turned out to pasture; and (4) October of Year 2, at weaning. Initial liver samples were collected from 45 cows in the INORG group and 43 cows from the ORG group. Due to culls, death loss, and insufficient sample amount, the number of liver samples analyzed for each time period differed (range = 35 to 45 liver samples per treatment per collection period).

Liver biopsies were also collected from a subsample of calves (*n* = 78) at the following two time points: (1) May of Year 2; initial biopsy collected when calves averaged 53 days of age, and (2) October of Year 2 at weaning when calves averaged 202 days of age. Analysis of liver mineral content was performed by the Michigan State University Diagnostic Center for Population and Animal Heath (Lansing, MI). Tissues were dried overnight in a 75 °C oven and then digested overnight in approximately 10× the dry tissue mass of nitric acid. The digested samples were diluted with water to 100× the dried tissue mass. Elemental analysis was performed according to the method by Wahlen et al. [16] using an Agilent 7500ce Inductively Coupled Plasma—Mass Spectrometer (ICP/MS; Agilent Technologies Inc, Santa Clara, CA, USA).

### 2.3. Cow Reproductive Performance

Thirty days following the initiation of mineral supplementation in Year 1, cows from both treatment groups were bred to one of two Angus sires using the prostaglandin six-day controlled internal drug release (CIDR) timed AI protocol [17]. In Year 2, cows were bred to a single Angus bull using a 7-day CIDR timed AI protocol [18]. In both years, Angus clean-up bulls were turned out 10 days after AI to cover any cows that did not conceive to AI. Clean-up bulls remained with cows for 60 days. Pregnancy was determined each year via trans-rectal ultrasonography, and crown–rump length was used to determine fetal age. Data were analyzed to determine the influence of mineral supplementation on first-service AI conception rate and breeding season pregnancy rate.

### 2.4. Health Management

During the experiment, cowherd health was managed according to established protocols for the Antelope Research Station. Prior to breeding, cows were vaccinated against infectious reproductive pathogens with a modified live IBRV/BVDV/PI_3_/Leptospirosis/Campylobacter vaccine. All calves were vaccinated against Clostridial organisms in May of Year 2, and revaccinated against viral respiratory pathogens (IBRV, BRSV, PI_3_, and BVDV types I and II), Mannheimia hemolytica, Histophilus somni, and Clostridial organisms 3 weeks prior to weaning in September of Year 2 with booster vaccinations administered in October of Year 2. Parasiticide (doramectin; Dectomax^®^, Zoetis, Florham Park, NJ USA) treatments were given to cows and calves in the summer and again in October of Year 2.

### 2.5. Passive Immunity Transfer Status

Transfer of passive antibodies was assessed by measuring total protein levels in sera samples taken from calves at 24–72 h of age obtained via spectrophotometric methods using the VetAce Clinical Chemistry System (Alfa Wasserman Diagnostic Technologies, West Caldwell, NJ, USA). A subsample of 25 serum samples taken from calves in each treatment group was collected to provide sufficient power to detect a difference of 0.5 mg/mL between treatment groups.

### 2.6. Vaccine Titers

Antibody titers to infectious bovine rhinotracheitis virus (IBRV), bovine respiratory syncytial virus (BRSV), and bovine viral diarrhea virus (BVDV) were ascertained and compared between groups, using the inverse log titer for comparison. The methods used for the analysis of IBRV and BVDV types I and II can be found in the OIE Terrestrial Manual [19,20] respectively, and BRSV was determined using the method described by Fulton et al. [21]. A subsample of 80 calves had blood collected at initial vaccination and 3 weeks later at weaning prior to booster vaccination. A randomly selected subsample of 40 sera samples from calves in each treatment group was collected to provide sufficient power to detect a mean difference of 2 logs between groups.

### 2.7. Viral Shedding Status

Viral shedding statuses of IBRV, BVDV, and BRSV were determined via PCR analysis using an ABI 7500 real-time PCR system (Applied Biosystems, Waltham, MA, USA) of deep nasal swabs collected in September of Year 2, 3 weeks prior to weaning. Determination of viral shedding statuses for IBRV, BVDV types I and II, and BRV were performed according to methods used by Moore et al., Mahlum et al., and Boxus et al. [22,23,24]. A randomly selected subsample of 100 samples from calves in each treatment group was collected to provide sufficient power to detect a difference of 20% prevalence of shedding between groups.

### 2.8. Statistical Analysis

Cow and calf liver mineral status, cow body weights, cow BCS and passive immunity, and calf vaccine response were all analyzed via the repeated measures model using the PROC MIXED procedure of SAS [25]. The effect of treatment was analyzed using animal within treatment as the error term, and effects of time and any interaction were analyzed using the residual as the error term. All covariance structures were modeled initially. The indicated best fit model of TOEP was used as the covariance structure. Cow age at the initiation of the trial was used as a covariate for BW and BCS. Animal served as the experimental unit. Calf weaning weight was analyzed using the PROC MIXED procedure of SAS. Calf age and sex were included as covariates. When the F statistic was significant (*p* < 0.05), mean separation was performed using least significant differences.

Since pregnancy status (AI bred—yes or no, and pregnancy status—pregnant or not pregnant) is a binomial distribution, the proportion (number observed in the class divided by number in the treatment) of cows in each treatment classification was analyzed as a binomial distribution in the GLIMMIX procedure of SAS. Each year was analyzed separately, as Year 1 cows were on treatment for 30 days prior to the start of the breeding season, and Year 2 cows were on treatment for 394 days before the start of the breeding season. The statistical model included treatment, dam age, dam BCS at the start of the breeding season, and days postpartum at AI. Interactions with a significance value of *p* > 0.20 were removed from the complete model in a stepwise manner to derive the final reduced model for each variable. Differences were considered to be significant when *p* ≤ 0.05.

## 3. Results and Discussion

### 3.1. Cow Body Weight and Body Condition Score

**Figure 1:**
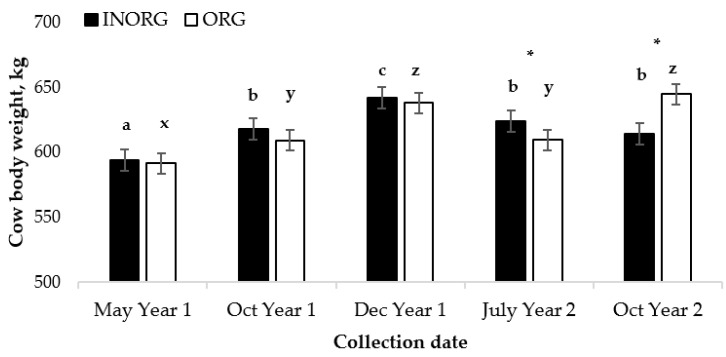
Interaction between Cu and Zn source and collection date (*p* < 0.0001) for cow body weight ^1,2^. ^1^ Cow age was used as a covariate in the model and was significant (*p* < 0.0001). ^2^ Cows had ad libitum access to a mineral supplement that provided 15 mg Cu and 15 mg Zn/kg diet DM as either CuSO_4_ and ZnSO_4_ (INORG) or as Bioplex Cu 10% and Bioplex Zn 15% (ORG) from 30 days prior to the breeding season through weaning. Calves had ad libitum access to the same mineral supplement as their dam from birth through weaning. ^a,b,c^ Means lacking common superscripts differ (INORG) (*p* < 0.0100). ^x,y,z^ Means lacking common superscripts differ (ORG) (*p* < 0.0074). * Treatment means within month differ (*p* < 0.0346).

An interaction between the treatment and the time was observed for cow BWs (*p <* 0.0001; Figure 1). Initial cow BW did not differ between the treatments. However, in July of Year 2, the cows from the INORG treatment group were heavier (*p* < 0.05) than the cows in the ORG treatment group, but at the end of the trial, the cows in the ORG treatment group had heavier (*p* < 0.0001) BWs than the cows in the INORG treatment group. The body weights of the cows in both treatment groups increased during the grazing season in Year 1 (*p* < 0.0001 and *p* < 0.01 for INORG and ORG, respectively) and continued to increase from October of Year 1 to December of Year 1 (*p* < 0.001 and *p* < 0.0001 for INORG and ORG, respectively).

From December of Year 1 to July of Year 2, cow BW decreased in both treatment groups (*p* < 0.01 and *p* < 0.0001 for INORG and ORG, respectively); however, the cows in the ORG treatment group had a greater decrease in BW than the cows in the INORG treatment group during this time period *(p* < 0.05).

There was no change in the BWs of the cows in the INORG treatment group from July of Year 2 to October of Year 2 (*p* = 0.15), while the BWs of the cows receiving the ORG supplement increased (*p* < 0.0001).

**Figure 2:**
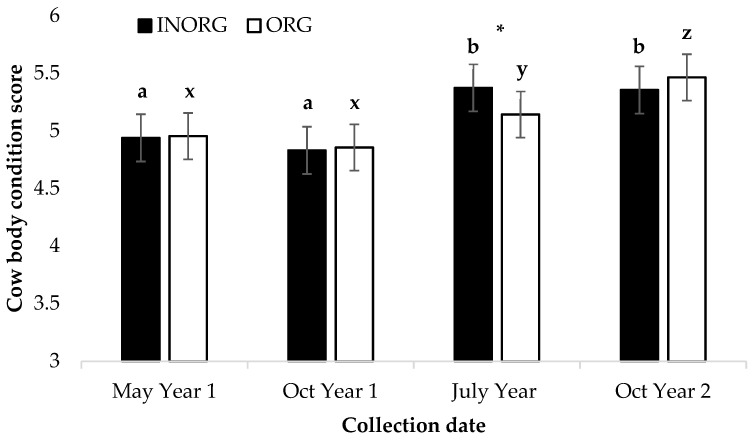
Interaction between Cu and Zn source and collection date (*p* = 0.0203) for cow body condition score ^1,2,3^. ^1^ Cow age was used as a covariate in the model but was not significant (*p* > 0.05). ^2^ Cows had ad libitum access to a mineral supplement that provided 15 mg Cu and 15 mg Zn/kg diet DM as either CuSO_4_ and ZnSO_4_ (INORG) or as Bioplex Cu 10% and Bioplex Zn 15% (ORG) from 30 days prior to the breeding season through weaning. Calves had ad libitum access to the same mineral supplement as their dam from birth through weaning. ^3^ Cow body condition score (BCS) was measured from 1 to 9; 1 = extremely emaciated, 9 = very obese. ^a,b^ Means lacking common superscripts differ (INORG) (*p* < 0.0001). ^x,y,z^ Means lacking common superscripts differ (ORG) (*p* < 0.0218). * Treatment means within month differ (*p* < 0.0047).

A treatment by time interaction was observed for the source of minerals and the collection date for cow BCSs (*p* < 0.05; Figure 2). Cow BCSs were similar in May and October of Year 1 for both treatments. From October of Year 1 to July of Year 2, the BCSs increased in both treatments (*p* < 0.001); however, the increase was greater in the cows in the INORG treatment group than in the ORG treatment group (*p* < 0.01). The body condition score continued to increase from July of Year 2 to October of Year 2 for the cows in the ORG treatment group (*p* < 0.0001), but not for the cows in the INORG treatment group.

### 3.2. Cow Liver Zn and Cu

**Figure 3:**
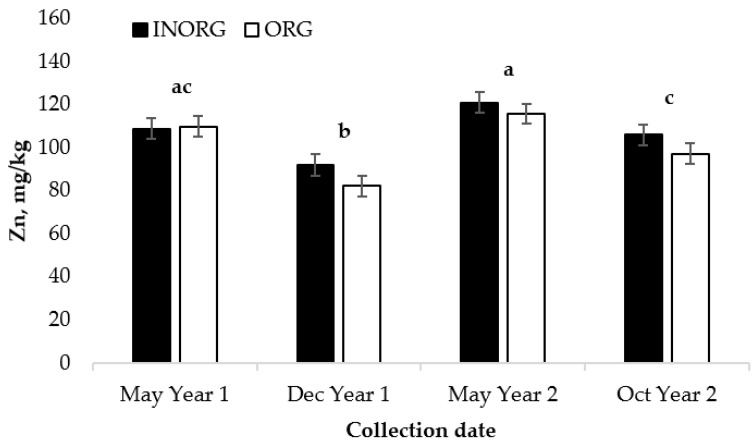
Influence of treatment and collection date on concentration of liver Zn in cows ^1,2,3^. ^1^ Cows had ad libitum access to a mineral supplement that provided 15 mg Cu and 15 mg Zn/kg diet DM as either CuSO_4_ and ZnSO_4_ (INORG) or as Bioplex Cu 10% and Bioplex Zn 15% (ORG) from 30 days prior to the breeding season through weaning. ^2^ May Year 1, start of trial; Dec Year 1, cows’ primary forage is hay; May Year 2, cows return to pasture with calves; Oct Year 2, end of trial, calves weaned. ^3^ Collection date, *p* < 0.0001; treatment, *p* = 0.2668; treatment x collection date, *p* = 0.3998. ^a,b, c^ Means lacking common superscripts differ *p <* 0.05.

The cow liver Zn concentration was not affected by the treatment (*p* = 0.27), but the concentrations did change over time (*p* < 0.0001; Figure 3). While on pasture, the liver Zn concentrations decreased in the cows in both Year 1 and Year 2 (*p* = 0.0505). However, the liver Zn concentrations increased when the commercial crude protein supplement was fed over the winter months (*p* < 0.0001).

**Figure 4:**
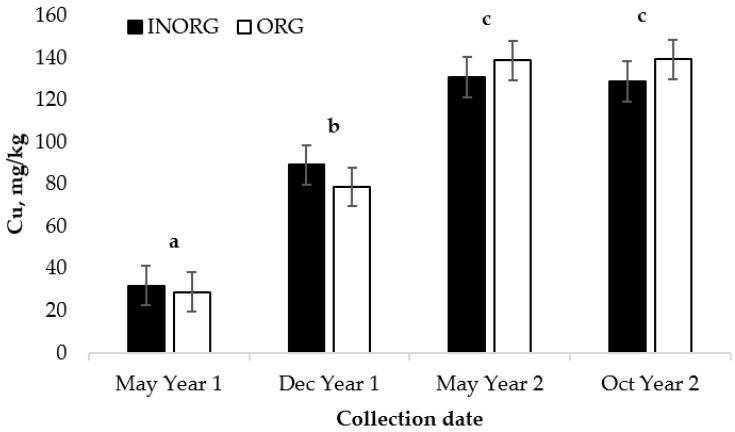
Influence of treatment and collection date on concentration of liver Cu in cows ^1,2,3^. ^1^ Cows had ad libitum access to a mineral supplement containing 15 mg/kg Cu provided as either CuSO_4_ or Bioplex Cu 10% and 15 mg/kg Zn as either ZnSO_4_ or Bioplex Zn 15% from 30 days prior to the breeding season through weaning. ^2^ May Year 1, start of trial; Dec Year 1, cows’ primary forage is hay; May Year 2, cows return to pasture with calves; Oct Year 2, end of trial, calves weaned. ^3^ Collection date, *p* < 0.0001; treatment, *p* = 0.9040; treatment x collection date, *p* = 0.4184. ^a,b,c^ Means lacking common superscripts differ (*p* < 0.05).

There was no influence of the treatment on the concentration of Cu in the liver (*p* = 0.90). However, when the cows were supplemented with minerals from either treatment, the liver Cu concentration increased from the initial liver biopsy date (May of Year 1) until the biopsy in May of Year 2 (*p* < 0.0001; Figure 4). The liver Cu concentrations did not differ between the May Year 2 and October Year 2 samples (*p* = 0.99).

### 3.3. Cow AI Conception Rates

**Figure 5:**
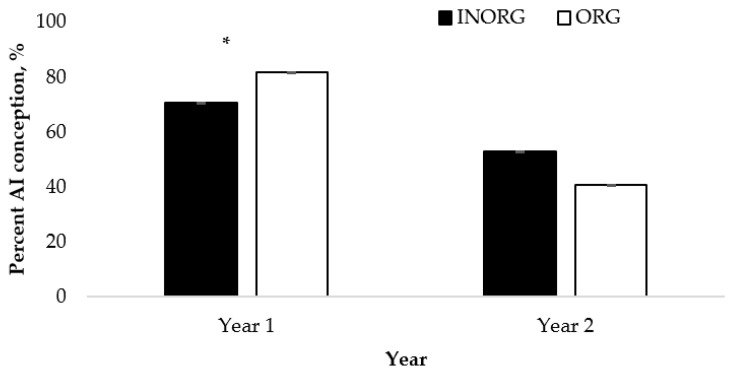
Interaction between Cu and Zn source and year (*p* = 0.0032) for artificial insemination conception rate of cows ^1,2^. ^1^ Dam BCS, age, and number of days postpartum were included as covariates; the number of dam days postpartum was significant (*p* = 0.0006). ^2^ Cows had ad libitum access to a mineral supplement that provided 15 mg Cu and 15 mg Zn/kg diet DM as either CuSO_4_ and ZnSO_4_ (INORG) or as Bioplex Cu 10% and Bioplex Zn 15% (ORG) from 30 days prior to the breeding season through weaning. Calves had ad libitum access to the same mineral supplement as their dam from birth through weaning. * Treatment means differ within year (*p* < 0.0209).

There was an interaction between the source of Cu and Zn and the year for the AI conception rate (*p* < 0.01; Figure 5). The cows in the ORG treatment group had greater AI conception rates than the cows from the INORG treatment group in Year 1 (*p* < 0.05), but there was no difference in Year 2 (*p* = 0.0624). In Year 1, the number of days postpartum also influenced the AI conceptions, with the conception rates increasing (*p* < 0.05) for the cows less than 40 days postpartum (55%) compared to 80 to 100 days postpartum (78%). There was no difference observed for overall pregnancy for either the year or the main effect of treatment (*p* < 0.05).

### 3.4. Calf 205-day Adjusted Weaning Weight

The calves in the ORG treatment group had a heavier (*p* < 0.05; Table 5) 205-day adjusted weaning weight than the calves from the INORG treatment group. As expected, the steers had heavier (*p* < 0.0001) 205-day adjusted weaning weights than the heifers.

### 3.5. Transfer of Passive Immunity and Calf Response to Vaccine

There was not a difference in the transfer of passive immunity from the cows to the calves (Table 5) as determined via the total protein levels in the sera samples taken from the calves at 24–72 h of age between treatments (*p* = 0.7950) or sexes (*p* = 0.7580). The results for the calf vaccine response for BRSV, BVDV types I and II, and IBRV are shown in Table 5. No differences were observed for BRSV for the main effect of treatment (*p =* 0.3995), collection period (*p =* 0.0887), or sex (*p =* 0.9127). Similarly, there were no differences observed for IBRV for the main effect of treatment (*p =* 0.9146), collection period (*p =* 0.4257), or sex (*p =* 0.8803). There was a collection period difference observed for BVDV type I (*p =* 0.0020) and BVDV II (*p =* 0.0007), respectively.

### 3.6. Calf liver Zn and Cu

**Figure 6:**
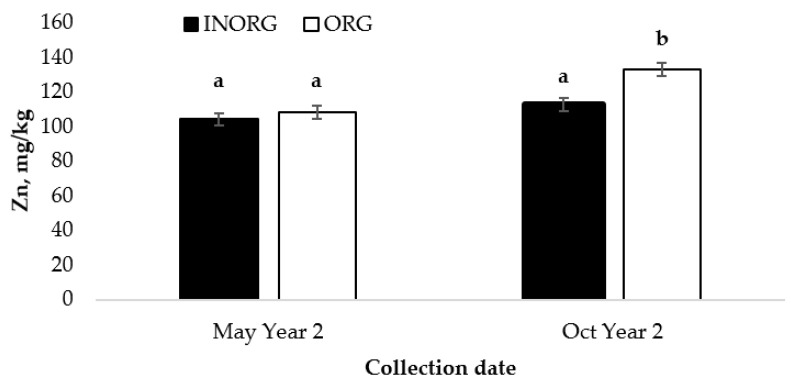
Interaction between Cu and Zn source and collection date (*p* = 0.0139) for liver Zn concentration in suckling calves ^1,2^. ^1^ Cows had ad libitum access to a mineral supplement that provided 15 mg Cu and 15 mg Zn/kg diet DM as either CuSO_4_ and ZnSO_4_ (INORG) or as Bioplex Cu 10% and Bioplex Zn 15% (ORG) from 30 days prior to the breeding season through weaning. Calves had ad libitum access to the same mineral supplement as their dam from birth through weaning. ^2^ May Year 2 = initial liver biopsy collected from calves; Oct Year 2 = calves weaned, and final biopsy collected. ^a,b^ Means lacking common superscripts differ (*p* < 0.0001).

Liver biopsies were collected from the calves in May and October of Year 2. There was an interaction observed between the treatment and collection date for liver Zn concentrations in the calves (*p* < 0.05; Figure 6). Calves from the ORG treatment group had an increase (*p* < 0.0001) in the liver Zn concentration from the initial biopsy to the final biopsy, while no difference (*p* = 0.07) was detected in the calves from the INORG treatment group between these time points.

**Figure 7:**
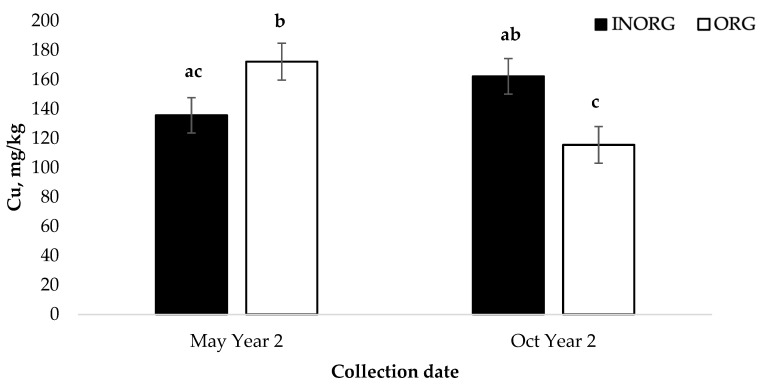
Cu and Zn source by collection date interaction (*p* = 0.0002) on the liver Cu concentrations in suckling calves ^1,2^. ^1^ Cows had ad libitum access to a mineral supplement that provided 15 mg Cu and 15 mg Zn/kg diet DM as either CuSO_4_ and ZnSO_4_ (INORG) or as Bioplex Cu 10% and Bioplex Zn 15% (ORG) from 30 days prior to the breeding season through weaning. Calves had ad libitum access to the same mineral supplement as their dam from birth through weaning. ^2^ May Year 2 = initial liver biopsy collected from calves; Oct Year 2 = calves weaned, and final biopsy collected. ^a,b,c^ Means lacking common superscripts differ (*p* < 0.0365).

An interaction between the treatment and the collection date was also observed for the liver Cu concentrations in the calves (*p* < 0.001; Figure 7). At the initial biopsy in May of Year 2, the liver Cu concentrations were greater in the calves from the ORG treatment group than in the calves from the INORG treatment group (*p* < 0.05). However, at the final biopsy in October of Year 2, the calves in the INORG treatment group had greater liver Cu concentrations than those in the ORG treatment group (*p* < 0.01).

## 4. Discussion

### 4.1. Cow Liver Zn and Cu

For the cow liver Zn concentrations, the periods when the Zn levels were lowest coincided with gestation. During this time, there would be a large draw of Zn required for the development of epithelial tissue (e.g., the placenta and fetus). Andrieu et al. [26] reported that Zn plays a key role in maintaining the structural integrity of tissues, such as the epithelial tissue of the reproductive tract. Hansard et al. [27] also indicated that as the fetus grows, the Zn concentrations increase in bovine conception products (placenta, placental fluids, and fetus). The cows began calving in March of Year 2, which could allow for enough time for the liver Zn concentrations to be repleted and result in an increase by May of Year 2. The decrease between May of Year 2 and October of Year 2 could again be attributed to the fact that the cows had been re-bred and placental and fetal growth were utilizing the available Zn. 

The increase in liver Cu concentration observed in this study as a result of mineral supplementation was demonstrated in previous research [28,29]. The initial liver Cu concentrations in this experiment would be considered marginally deficient [30]. As the amount of time that the cows consumed fortified minerals progressed, it was not unexpected that the liver Cu scores would increase. The plateau in liver Cu concentration may be due to the sulfur concentration in the water and the greater amount of Mo present in the forage in Year 2. In combination with S, Mo is a potent antagonist of Cu [8,31,32], and it is possible that the level of supplemental Cu in this experiment was not sufficient enough to overcome the antagonism.

### 4.2. Cow Body Weight and Body Condition Score

The calves in this study were weaned in October of Year 1 when the BWs of the cows were collected, thus removing the demand for lactation. The end of lactation coupled with the addition of a growing fetus is expected to contribute to an increase in the BW, as indicated by these results. From December of Year 1 to July of Year 2, the cows transitioned from late gestation to early lactation. Both instances require elevated energy demands that can cause a decrease in the BW, as indicated by the results.

With no differences between the liver Cu and Zn concentrations between the treatments in this study, it is not clear why the differences in BW occurred between July of Year 2 and October of Year 2. Ahola et al. [28] reported an effect of the time period on cow BW when comparing cows supplemented with inorganic (100% inorganic) or mixed (50% organic/50% inorganic) sources of Cu, Zn, and Mn to a control (non-supplemented) group, but did not observe any differences among the treatments. They reported that cow weights decreased following calving, but returned to previous levels by the middle of summer [28]. This discrepancy between Ahola et al. [28] and the present study may be due to the fact that the cows were not weighed at similar time points. The decrease in the BW following calving reported by Ahola et al. [28] is likely associated with the removal of the fetus and the associated placental tissue following birth. It was stated that the cow BWs were collected at 56-day intervals, but researchers did not indicate where that interval fell relative to parturition. In the current study, the cows were not weighed until the middle of the summer following calving. A decrease in BW may have also occurred after calving, but was not recorded, as the BW was not measured at that time point. Another cause for the contrast between the results of Ahola et al. [28] and the current study may be the duration of supplementation prior to calving. Ahola et al. [28] supplemented cows for 82 days (Year 1) and 81 days (Year 2) prior to the average calving date, whereas the cows in the current study were supplemented for 283 ± 19 days prior to the average calving date. Zinc plays a role in maintaining the structural integrity of tissues, such as the epithelial tissue found in the reproductive tract that is shed from the body at parturition [26]. Hansard et al. [27] indicated that as the fetus grows, the Zn concentrations increase in the bovine conception products (placenta, placental fluids, and fetus). The body can become Zn deficient when the metabolism directs stored Zn to the reconstruction of tissues that were damaged from parturition. Furthermore, if cows become Zn deficient following parturition, it can cause the cows to lose weight, because Zn plays a role in muscle turnover. Engle et al. [33] demonstrated that Zn plays a critical role in the proteolytic enzyme systems associated with muscle protein turnover. The muscle protein accretion decreased when the animals were not supplemented with Zn, and once the supplement was provided again, the muscle protein accretion returned to normal levels within 14 days. Thus, if the cows were deficient in Zn and the amount of muscle turnover was decreased, it would be expected for the cows to begin losing weight. 

As previously discussed, the cows in the INORG treatment group had heavier BWs than the cows in the ORG treatment group, which could be a result of the differences in BCSs between treatments observed in July of Year 2 in this study. 

In contrast to these results, Ahola et al. [28] observed no differences in the BCS between cows supplemented with either inorganic or organic trace minerals. Further, Nayeri et al. [34] reported no change in BCS when dairy cows were assigned to one of the following treatment groups 28 ± 15 days prior to calving: (1) 75 mg of supplemental Zn/kg of DM, provided entirely as zinc sulfate; (2) 33.3 mg of Zn sulfate/kg of DM in the prepartum and 15.5 mg of Zn sulfate/kg of DM in the postpartum diet was replaced by Availa Zn (organic form); and (3) 66.6 mg of Zn sulfate/kg of DM in the prepartum diet and 40.0 mg of Zn sulfate/kg of DM in the postpartum diet was replaced by Availa Zn (organic form). The lack of difference in BCS observed by Nayeri et al. [34] compared to the current study may be due to the fact that the amount of zinc available in all of the treatment diets surpassed the NRC [35] requirement for both the pre- and postpartum dairy cows. In the current study, the cows in the organic treatment group were consuming a supplement that was only composed partially of Zn in the organic form, which could have contributed to the inconsistency in the BCS results. The cows may not have been receiving as much readily available Zn as the organic treatment provided in the current study. Another factor contributing to the inconsistency in the BCS results between Nayeri et al. [34] and the current study is the difference in breeds. Du et al., Gooneratne et al., Littledike et al., Mullis et al., Ward et al., and Wiener et al. [36,37,38,39,40,41] all reported that different breeds of cattle and sheep have different requirements for certain trace minerals. The specific cause for the different requirements among breeds has yet to be elucidated but may be related to the retention or excretion rates, growth rate, or milk production. Finally, differences in the length of supplementation prior to calving (28 days versus 283 days) and the timing of BCS observation can contribute to contrasting results. In the current experiment, the cows had access to their treatments from 30 days prior to breeding through the subsequent weaning. By comparison, Ahola et al. [28] began supplementation either 81 or 82 days prior to calving and continued until either 110 or 135 days after calving.

### 4.3. Cow AI Conception Rates

The AI conception results observed in this study agree with those observed by Stanton et al. [42], who detected an improvement in the AI pregnancy rate when an organic mineral source was provided to the cows. The researchers provided the cows with the following three levels of Cu, Zn, Mn, and Co: (1) low-level inorganic, (2) high-level inorganic, and (3) high-level organic. The cows were of similar genetic makeup, and the study was conducted in a similar environment to the present study. These researchers reported that the cows provided with the high-level organic treatment had an increased AI conception rate. In contrast, Muehlenbein et al. [29] compared first-calf cows provided with an organic Cu source (110–150 mg Cu/d) or inorganic (210–300 mg Cu/d) source with non-supplemented controls (50–60 mg/d Cu) over a two-year period. In Year 1, Muehlenbein et al. [29] reported a greater overall pregnancy percentage in the control group (non-supplemented) when compared to the inorganic treatment group, but found no difference between the control and organic supplementation groups. There were also no differences observed for the pregnancy percentage between the inorganic and organic treatments in the first year. However, in Year 2, the cows in the organic treatment group had a greater percentage of overall pregnancy within the first 30 days compared with those in the control treatment group; however, no differences were reported for the overall pregnancy rate between the organic and inorganic supplementation groups or the inorganic and control groups in Year 2.

### 4.4. Calf Liver Zn and Cu

The results for the calf liver Zn concentrations indicate that Zn from an organic source may be more bioavailable to the body for absorption and storage. These results agree with those of previous research, where Zn provided as either Zn methionine or Zn proteinate was shown to accumulate more readily in multiple tissues in cattle [43]. Spears [5] reported that organic Zn sources are usually more soluble in the rumen than inorganic sources. In a study by Spears and Kegley [44], steers that were fed proteinated Zn had a greater ruminal soluble Zn concentration during the growing and finishing phases compared to the steers supplemented with Zn oxide. Increased solubility should increase the amount of Zn presented to the intestinal epithelium that is available for absorption. The increase in dietary Zn could be a result of either an increased Zn concentration in milk or an increased consumption of the mineral supplement by the calves.

The interaction between the treatment and collection date observed for the calf liver Cu concentration is perplexing, as it is reasonable to expect Cu from organic sources to be more available for absorption than Cu from inorganic sources. However, the liver Cu stores in the dams of these calves was not affected by the treatment. This suggests that sources of Cu may be metabolized differently after absorption, and that organic Cu sources may be transported across the placenta more effectively than Cu from inorganic sources. The reason for lower liver Cu concentrations at weaning in calves from the ORG group is also unclear. If the Cu is more available for absorption, the liver concentrations would be expected to remain stable, if not increase during supplementation. Once again, differences in the post-absorption metabolism may be responsible for the differences.

### 4.5. Calf 205-day Adjusted Weaning Weight

The greater weaning weight (WW) observed in this study may be due to the enhanced availability of organic Zn and Cu to the body, increasing the amount of protein turnover, while not changing the rate of protein degradation.

Engle et al. [33] demonstrated that Zn plays a critical role in proteolytic enzyme systems associated with muscle protein turnover. The muscle protein accretion decreased when the animals were not supplemented with Zn. These results agree with those from the work by Stanton et al. [42] in which calves that were fed organic minerals had heavier WWs than calves that were fed a high level of inorganic trace minerals. Stanton et al. [42] indicated that feeding calves high levels of inorganic mineral when Fe is present had negative effects on calf WW. Furthermore, the study previously described by Ahola et al. [28] reported that calves in the organic treatment group had a greater 205-day WW than the calves from the inorganic treatment group in Year 1, but not in Year 2. However, the calves from Ahola et al. [28] that were in the control (non-supplemented) group had a greater 205-day WW than the supplemented calves regardless of the mineral source for Years 1 and 2. It is not clear why this result occurred.

### 4.6. Transfer of Passive Immunity and Calf Response to Vaccine

The previously described study by Muehlenbein et al. [29] reported similar findings for passive immunity observed in this study. Muehlenbein et al. [29] analyzed the IgG titer concentration in the serum collected from calves 24–36 h after calving. In both Year 1 and Year 2 the calves from the organic treatment group had a greater concentration of IgG titers present in the blood serum than the calves from the control treatment group; however, no differences were detected between the inorganic and organic treatments.

As expected, the titer values in this study increased from pre-vaccination to post-vaccination for both BVDV I and BVDV II. These results agree with those of previous research by Chang et al. [45].

Research investigating mineral supplementation in general, and the effect it has on viral shedding, is limited. In the current study, no differences were detected between the calves provided with ORG Cu and Zn compared to those provided with INORG Cu and Zn. Out of all the observations, only one steer and one heifer from the INORG treatment group tested positive for bovine coronavirus (BCV). With this limited number of positive samples, a statistical analysis was not possible; therefore, no results are shown. Orr et al. [46] conducted four trials investigating changes in blood serum Cu and Zn concentrations during transit stress and/or stress, and when the calves were challenged with IBRV. The calves were supplemented with a trace mineral premix, of an unknown source, which was formulated to provide Zn, 50 ppm; Mn, 10 ppm; Fe, 50 ppm; Cu, 5 ppm; and Co, 0.1 ppm. The researchers reported an increase in the urinary Cu and Zn excretion in the cattle inoculated with IBRV. These researchers also observed that the blood serum concentration of Zn decreased in morbid or IBRV-challenged calves, while the Cu concentration increased, indicating that health status affects Cu and Zn levels. Therefore, the lack of differences in viral shedding between calves supplemented with organic or inorganic sources of Cu and Zn pre- and post-gestation in the current study may not have been observed because the calves were provided with Cu and Zn, regardless of the source.

## 5. Conclusions

Supplementing cows throughout gestation with organic Cu and Zn improved the initial AI conception rate and the final cow BW. Mineral supplementation, regardless of source, improved the BCS. The calves produced from the cows supplemented with organic Zn had greater concentrations of Zn present in the liver compared to the calves from the cows supplemented with inorganic Zn. However, the calves from the cows supplemented with inorganic Cu had a greater concentration of Cu present in the liver compared to the calves from the cows supplemented with organic Cu. Supplementing the calves with organic Cu and Zn resulted in increased weaning weights compared to the supplementation of Cu and Zn in the inorganic form. Collectively, these data suggest that supplementation with organic minerals may be beneficial to a cow–calf producer that is marketing calves at weaning.

## Figures and Tables

**Table 1:** Pasture nutrient composition ^1,2^.

Item	Treatment			
	INORG ^3^		ORG ^4^	
	Year 1	Year 2	Year 1	Year 2
CP, %	9.40	8.78	9.10	8.47
ADF, %	37.13	33.72	38.58	32.98
NDF, %	63.14	68.92	65.69	67.81
Ash, %	7.70	7.50	7.29	7.44
Ether extract, %	2.43	2.20	2.34	1.99
Ca, %	0.28	0.38	0.32	0.36
P, %	0.19	0.17	0.18	0.18
Mg, %	0.11	0.13	0.11	0.11
K, %	1.57	1.55	1.39	1.32
S, %	0.18	0.17	0.16	0.17
Na, %	0.03	0.03	0.02	0.01
Cl, %	0.51	0.36	0.41	0.24
Se, mg/kg	0.48	0.66	0.61	0.29
Mn, mg/kg	50.33	48.00	46.50	38.33
Zn, mg/kg	40.00	26.50	36.25	25.67
Cu, mg/kg	18.33	11.75	19.25	14.00
Fe, mg/kg	222.67	221.75	309.25	230.67
Mo, mg/kg	0.98	4.35	1.10	3.02

^1^ Forage samples were collected at the beginning of the grazing period and averaged for each pasture in a rotational grazing system in each year. ^2^ All values are expressed on a DM basis. ^3^ INORG: Cows and calves had ad libitum access to a mineral supplement that provided 15 mg Cu and 15 mg Zn/kg of diet DM as CuSO_4_ and ZnSO_4_, respectively. ^4^ ORG: Cows and calves had ad libitum access to a mineral supplement that provided 15 mg Cu and 15 mg Zn/kg as Bioplex Cu 10% and Bioplex Zn 15%, respectively (Alltech, Inc., Nicholasville, KY, USA).

**Table 2:** Nutrient composition of hay ^1^ and winter supplement ^2^.

Nutrient	Hay (Source 1)	Hay (Source 2)	30% Crude Protein Supplement
CP, %	7.69	11.74	30.72
ADF, %	39.56	43.43	15.01
NDF, %	59.69	60.56	22.73
Ash, %	9.78	9.75	7.39
Ether, %	3.32	1.96	2.38
Ca, %	0.33	1.16	0.61
P, %	0.14	0.20	0.88
Na, %	0.21	0.05	0.37
Cl, %	0.45	0.13	0.45
Mg, %	0.17	0.23	0.45
K, %	1.09	1.93	1.48
S, %	0.35	0.16	0.55
Se, mg/kg	0.26	0.42	0.92
Fe, mg/kg	196	695	203
Cu, mg/kg	14	15	7
Mn, mg/kg	79	65	115
Zn, mg/kg	29	26	87
Mo, mg/kg	2.17	1.75	1.28

^1^ All cows received both sources of hay from immediately prior to calving through 1 day after calving. ^2^ All values are expressed on a DM basis.

**Table 3:** Nutrient composition of mineral supplements ^1^.

	Treatment	
Nutrient	INORG ^2^	ORG ^3^
Ca, %	13.40	13.40
P, %	12.05	12.1
Mg, %	1.56	1.55
K, %	0.42	0.43
Co, mg/kg	30.5	30.5
Cu, mg/kg	2340.0	2340.0
I, mg/kg	180	180
Mn, mg/kg	238.6	238.5
Zn, mg/kg	2340.0	2347.1
Vitamin A, IU/kg	441,000	441,000
Vitamin D_3_, IU/kg	33,075	33,075
Vitamin E, IU/kg	1102.5	1102.5

^1^ All minerals were supplemented in their inorganic form unless otherwise noted. ^2^ INORG: Cows and calves had ad libitum access to a mineral supplement that provided 15 mg Cu and 15 mg Zn/kg of diet DM as CuSO_4_ and ZnSO_4_, respectively. ^3^ ORG: Cows and calves had ad libitum access to a mineral supplement that provided 15 mg Cu and 15 mg Zn/kg as Bioplex Cu 10% and Bioplex Zn 15%, respectively (Alltech, Inc., Nicholasville, KY, USA).

**Table 4:** Estimated Cu and Zn intake and diet concentration for cows at various time points throughout the experiment ^1^.

	Intake				Diet Concentration			
	Cu, mg		Zn, mg		Cu, mg/kg Diet DM		Zn, mg/kg Diet DM	
Time Period ^2^	INORG ^3^	ORG ^4^	INORG	ORG	INORG	ORG	INORG	ORG
May Year 1	489.2	502.9	832.3	618.0	30.7	31.7	52.3	38.9
October Year 1	450.2	460.2	747.1	559.1	32.7	33.7	54.2	41.0
December Year 1	404.2	413.4	695.6	547.4	34.5	35.5	59.4	47.0
July Year 2	384.5	416.9	771.3	599.1	24.2	26.6	48.6	38.3
October Year 2	359.5	395.9	694.1	560.7	26.2	28.0	50.5	39.6

^1^ Estimates were developed based upon actual cow weights at each time point and predicted dry matter intake based on NASEM (2016). ^2^ Each value includes minerals contributed by grazed forage and treatment mineral supplement except Dec Year 1, which also includes minerals contributed by a commercial crude protein supplement. ^3^ Cows had ad libitum access to a mineral supplement that provided 15 mg Cu and 15 mg Zn/kg diet DM as either CuSO_4_ and ZnSO_4_ (INORG) from 30 days prior to the breeding season through weaning. Calves had ad libitum access to the same mineral supplement as their dam from birth through weaning. ^4^ Cows had ad libitum access to a mineral supplement that provided 15 mg Cu and 15 mg Zn/kg diet DM as Bioplex Cu 10% and Bioplex Zn 15% (ORG) from 30 days prior to the breeding season through weaning. Calves had ad libitum access to the same mineral supplement as their dam from birth through weaning.

**Table 5:** Least squares means for weaning weight, passive immunity transfer status, and vaccine response ^1^ to bovine respiratory syncytial virus (BRSV), infectious bovine rhinotracheitis virus (IBRV), and bovine viral diarrhea virus (BVDV) types I and II of progeny from dams supplemented with inorganic or organic sources of Cu and Zn.

	Treatment (Trmt) ^2^			Sex			Collection Period (CP) ^3^			*p*-Value ^4^		
	INORG	ORG	SEM	Heifers	Steers	SEM	Pre Vacc.	Post Vacc.	SEM	Trmt	Sex	CP
WW ^5^, kg	252.6 ^a^	259.1 ^b^	2.69	247.2 ^a^	264.5 ^b^	2.69	-	-	-	0.0167	<0.0001	-
Total protein ^6^, g/dL	7.36	7.43	0.275	7.44	7.36	0.278	-	-	-	0.7950	0.7580	-
BRSV, log ^2^	1.18	1.11	0.075	1.14	1.15	0.075	1.10	1.19	0.051	0.3995	0.9127	0.0887
IBRV, log ^2^	4.85	4.89	0.306	4.85	4.89	0.306	4.75	4.99	0.297	0.9146	0.8803	0.4257
BVDV type I, log ^2^	4.96	5.48	0.366	5.21	5.22	0.366	4.75 ^a^	5.68 ^b^	0.292	0.1610	0.9704	0.0020
BVDV type II, log ^2^	5.26	5.66	0.282	5.41	5.51	0.282	4.96 ^a^	5.96 ^b^	0.282	0.1529	0.7262	0.0005

^1^ Determined via antibody titers ascertained and compared between groups, using the inverse log titer for comparison. ^2^ Cows had ad libitum access to a mineral supplement that provided 15 mg Cu and 15 mg Zn/kg diet DM as either CuSO_4_ and ZnSO_4_ (INORG) or as Bioplex Cu 10% and Bioplex Zn 15% (ORG) from 30 days prior to the breeding season through weaning. Calves had ad libitum access to the same mineral supplement as their dam from birth through weaning. ^3^ Pre-vaccination collection = Sept Year 2; pre-vaccination collection = Oct Year 2. ^4^ Probability of difference among least squares means; ^5^ 205-day adjusted weaning weight. ^6^ Transfer of passive immunity determined via total protein levels in sera samples collected from calves at 24–72 h of age. ^a,b^ Means lacking common superscripts differ (*p* < 0.05).

## Data Availability

The data presented in this study are available upon request from the corresponding authors.
